# Social integration and mental health - a decomposition approach to mental health inequalities between the foreign-born and native-born in Sweden

**DOI:** 10.1186/s12939-019-0950-1

**Published:** 2019-04-03

**Authors:** Anna Brydsten, Mikael Rostila, Andrea Dunlavy

**Affiliations:** Department of Public Health Science, Centre for Health Equity Studies (CHESS), Stockholm University/Karolinska Institutet, SE-105 91 Stockholm, Sweden

**Keywords:** Mental health inequality, Foreign-born, Social integration, Oaxaca–Blinder decomposition, Sweden

## Abstract

**Background:**

The increasing mental health inequalities between native- and foreign-born persons in Sweden is an important public health issue. Improving social integration has been stressed as a key strategy to combat this development. While a vast amount of studies have confirmed the importance of social integration for good mental health, less is known about the role of different types of social integration, and how they relate to mental health inequalities. This study aimed to examine the extent to which indicators of social integration explained mental health inequalities between the native- and foreign-born.

**Methods:**

Based on the *Health on Equal Terms* survey from 2011/2015 in Västra Götaland, Sweden (*n* = 71,643), a non-linear Oaxaca–Blinder decomposition analysis was performed comparing native- and foreign-born individuals from Nordic-, European- and non-European countries. The *General Health Questionnaire* was used to assess psychological distress, while 11 items assessed employment conditions and economic disparities, social relations, and experiences of discrimination to measure different aspects of social integration.

**Results:**

Differences in social integration explained large proportions of observed mental health differences between the native- and foreign-born. Important indicators included low levels of social activity (20%), trust in others (17%) and social support (16%), but also labour market disadvantages, such as being outside the labour market (15%), unemployment (10%) and experiencing financial strain (16%). In analyses stratified by region of origin, low trust in others and discrimination contributed to the mental health gap between the native-born and European-born (17 and 9%, respectively), and the native-born and non-European-born (19 and 10%, respectively). Precarious labour market position was a particularly important factor in the mental health gap between the native-born and Nordic-origin (22%), and non-European origin (36%) populations.

**Conclusion:**

Social integration factors play a central role in explaining the mental health inequality between natives and migrants in Sweden. Our findings suggest that public health actions targeting mental health gaps could benefit from focusing on inequalities in social and economic recourses between natives and migrants in Sweden. Areas of priority include improving migrants’ financial strain, as well as increasing trust in others and social support and opportunities for civic engagement.

Recent evidence has suggested that mental health inequalities between native- and foreign-born persons in Sweden are increasing [1]. In order to combat this negative development, policy makers and public health researchers have proposed increasing social integration as a key strategy to reduce the prevalence of poor mental health among migrants [2–4]. Social integration is a multidimensional concept describing the capacity of people to participate in social, cultural, economic and political life in the community [5]. For migrants, who commonly face several social and economic adversities and challenges, lack of social integration may lead to increased stress and poor psychological well-being [6–9]. However, although several studies have linked migrants’ integration with health [10–14], most have examined the influence of only one or two dimensions of social integration, and have not directly assessed the extent to which different social integration factors might contribute to mental health inequalities between native- and foreign-born individuals. The present study aims to address this knowledge gap by using a decomposition analysis to assess the relative contribution of different social integration factors, such as labour market and economic adversities, social relations, and discrimination, in explaining mental health gaps between natives and groups of migrants in Sweden.

## Background

In recent years, the number of migrants has increased substantially in Sweden. In 2017, nearly 1.9 million people living in Sweden were foreign-born, corresponding to approximately every sixth person in the population [15]. With these sociodemographic changes, ensuring a healthy population is of high importance. Although migrants tend to show lower mortality rates relative to natives [16], they also tend to report poorer self-rated health, higher rates of long-term illness and poorer mental health, such as depression and anxiety [8, 12, 17–19]. The roots of mental health inequalities are often attributed to social integration issues, such as difficulty getting established in the labour market, social and economic deprivation, as well as social isolation and discrimination [2, 8, 20].

Employment has been the most studied aspect of social integration among migrants, as it entails active participation in society. Stable employment with good working conditions can provide multiple social and psychological benefits, including social interaction with co-workers, increased self-esteem, sense of identity, and daily time structures [21]. For migrants in particular, employment is also a key factor in resettlement in a new society, allowing for financial independence and sense of belonging. However, migrants in Sweden typically have higher rates of unemployment as well as temporary employment than natives [22]. They are also experience greater economic disadvantage as well as poorer psychosocial working conditions than the natives [23]. All of these factors have been associated with poor mental health among migrants [8, 13, 14, 24–26], as well as the general population [27–29]. Unemployment, precarious employment and economic deprivation may also act as moderating factors through socioeconomic circumstances of low education, poor working conditions and low income, leading to psychological distress. Due to migrants’ marginalised position on the labour market, unemployment may be particularly important social determinant of mental health inequality.

Labour market integration are also related to other dimensions of social integration, such as the formation of social relationships and networks. Social relationships may be particularly important for migrants’ mental health because they often have access to fewer social arenas compared to native-born [10, 30]. Studies of migrant mental health have noted the importance of social relationships and social networks in the country of residence, which can influence migrants’ ability to adapt and cope with their new social environment [3, 31]. Social relationships characterised by social and practical support and trust in others can promote social integration and well-being, reduce stress, and buffer against poor mental health [8, 14, 32, 33]. Participation in social activities, such as sports, gatherings with friends and family, and political and religious workshops, have been identified as activities that promote a good social environment, political and democratic platforms, and a foundation for trust in other people [32, 34], which in turn can promote social resilience and mental health [35]. In addition to the economic, labour market and social network disadvantages often seen among migrants, they also may face social integration barriers to a greater extent than natives. Previous studies have shown that incidents of physical or verbal harassment and discrimination among migrants’ have a deeply adverse impact on social integration [8], and an increased risk of poor mental health [36], low quality of life [34] and lower self-reported health status [37], particularly among non-white immigrant women [8, 38, 39].

These findings highlight the mental health importance of social integration in the destination country. Yet, it is likely that migrants’ degree of social integration and the relative importance of different social integration factors have a differential impact on mental health depending on country of origin [24]. For example, due to variation in experiences of stressful migration processes, language and cultural barriers, and exposure to threats, violence and discrimination, social integration and mental health is likely to vary within migrant populations [24, 34, 40]. It is therefore important to explore the magnitude of mental health inequalities between natives and different groups of migrants, and the differential impact of distinct types of social integration on such mental health inequalities. In this study, we used the Oaxaca–Blinder decomposition for non-linear models to investigate the relative contributions of variations in the distributions of social integration indicators as well as their differential effects in producing mental health inequalities between natives and groups of migrants in Sweden. With this approach, in comparisons to logistic regression models with interaction terms, we can quantify both the degree to which each indicator of social integration and the combined overall measure of social integration explain the mental health inequality between natives and migrants. The specific aim of this study was to assess 1) the extent to which different indicators of social integration (employment conditions, economic disparities, social relations, and experiences of discrimination) may explain differences in psychological distress between the native- and foreign-born, and 2) if the contribution of different indicators of social integration in the formation of mental health inequalities differs among groups of foreign-born (Nordic, European and non-European origin).

## Methods

### Data material

Cross-sectional data were obtained from the Health on Equal Terms (HET) surveys from 2011 and 2015 for Västra Götaland. The HET-survey is administered by the Public Health Agency of Sweden in collaboration with the region of Västra Götaland (i.e. Skaraborg, Älvsborg and Bohus county councils and parts of the City of Göteborg) and Statistics Sweden [41]. The national survey has been conducted annually since 2004, with additional samples for Västra Götaland in 2007, 2011 and 2015. The sample consisted of a random selection of individuals from age 16 to 84 from Västra Götaland. The participants answered a self-administered questionnaire covering areas such as health and well-being, health care, living habits, work and psychosocial working conditions, and social relationships. Data collection was conducted by mailed surveys with the option to answer via a web-based form. Register data from Statistics Sweden was also collected to assess age, gender, civil status, education, country of birth and citizenship.

In this study, pooled data was used from survey years 2011 and 2015. The response rate was 54.4% (*n* = 41,740) in 2011 and 55.5% (*n* = 52,348) in 2015. The majority of dropouts were registered as mail returns, while only a small fraction were due to not wanting to participate, questionnaire problems or inability to contact participants [41, 42]. Due to the exclusion of the non-working age population (i.e. younger than 18 and older than 65) the final sample of the pooled dataset was *n* = 71,643, of whom 7.9% (men *n* = 2581 and women *n* = 3099) were born outside Sweden.

### Measurements

#### Psychological distress

Psychological distress was measured by the 12-item version of the General Health Questionnaire (GHQ-12) which has been previously used in studies of migration and mental health [13, 43]. It is a well-validated screening instrument developed to assess non-psychotic mental illness, typically depressiveness and anxiety [44]. The participants were asked about their feelings and abilities during the last few weeks, such as enjoying day-to-day activities, being able to concentrate, making decisions, overcoming difficulties and problems, having sleeping difficulties, feeling unhappy, depressed, and losing confidence and self-worth [44]. The severity of each item was measured using a 4-point scale, dichotomised into ‘better than usual/as usual’ and ‘worse than usual/much worse than usual’. In accordance with a Swedish validation study [44], the items were summed into a 12 point summary index and then dichotomised, with three or more symptoms coded as having psychological distress.

#### Migration

Country of birth was obtained through the Total Population Register, and reported as origin from Africa, Asia, Europe (excluding the Nordic countries), North America, the Nordic countries (excluding Sweden), Oceania, Sweden and South America. The variable was then coded into people born in Sweden (referred to as natives) and those not born in Sweden (migrants). The group of migrants were then further categorised by region of origin; into Nordic countries, European countries (excluding Nordic countries), and non-European countries (i.e., Africa, Asia, Oceania, and North America and South America).

#### Social integration measures

The explanatory variables were selected in correspondence with previous research to capture the complex multidimensional process of social integration [10–14, 34]. An overview of all variables is shown in Table 1. All items were based on self-reported data, if not stated otherwise.

**Table 1:** Descriptive characteristics of native-born and foreign-born (origin from Nordic-, European- and non-European countries) in working age population (18–65 yrs.)

	Native-born (*n* = 65,963)		Foreign-born (*n* = 5680)			Nordic (*n* = 1589)			European (*n* = 2175)			Non-European (*n* = 1874)		
	%	(*n*=)	%	(*n*=)	*p*	%	(*n*=)	*p*	%	(*n*=)	*p*	%	(*n*=)	*p*
Survey (2011)	49.2	(32478)	73.4	(4167)	^*^	71.2	(1132)	^*^	73.7	(1602)	^*^	75.7	(1418)	^*^
Men	44.7	(29493)	45.4	(2581)		42.3	(672)		45.8	(996)		47.3	(887)	^*^
Married/cohabiting civil status	75.1	(49506)	81.0	(4603)	^*^	74.1	(1176)		81.8	(1780)	^*^	86.1	(1614)	^*^
Age, mean (sd)	45.3	(13.8)	44.5	(13.1)	^*^	52.6	(11.1)	^*^	43.7	(12.9)	^*^	39.5	(12.3)	^*^
Blue-collar occupational class	48.0	(31539)	61.4	(3236)	^*^	55.8	(880)	^*^	58.0	(1234)	^*^	70.4	(1300)	^*^
Compulsory education	15.9	(10424)	21.7	(1136)	^*^	24.8	(372)	^*^	14.0	(286)	^*^	28.4	(474)	^*^
Secondary education	54.4	(35710)	44.3	(2321)		52.1	(780)		44.8	(917)		36.8	(615)	
Post-secondary education	29.8	(19550)	34.1	(1785)		23.1	(346)		41.2	(844)		34.9	(583)	
Disposable income, mean (sd)^b^														
Q 1	48.1	(61.4)	43.6	(43.1)	^*^	30.6	(47.8)	^*^	45.0	(39.8)	^*^	49.0	(42.7)	^*^
Q 2	142.4	(15.1)	139.9	(15.2)		141.7	(15.4)		139.9	(15.1)		138.6	(15.2)	
Q 3	197.7	(15.8)	195.7	(15.5)		196.0	(15.8)		195.3	(15.4)		195.6	(15.4)	
Q 4	253.9	(18.8)	252.6	(18.5)		252.9	(18.4)		252.8	(18.9)		251.8	(18.6)	
Q 5	422.8	(1471.0)	340.0	(256.0)		387.4	(180.9)		403.1	(238.2)		421.2	(314.2)	
Employed/Self-employed	74.8	(46820)	57.6	(3024)	^*^	66.3	(961)	^*^	63.1	(1290)	^*^	44.1	(757)	^*^
Unemployed/Labour market practice	4.2	(2648)	11.0	(576)		5.3	(77)		9.4	(193)		17.6	(302)	
Outside the labour market ^a^	20.9	(13105)	31.4	(1651)		28.4	(412)		27.5	(561)		38.3	(657)	
Low support from co-workers	6.8	(4502)	11.4	(649)	^*^	6.4	(102)		10.8	(235)	^*^	16.1	(301)	^*^
Low support from manager	8.6	(5576)	10.1	(573)	^*^	7.2	(114)		9.9	(215)	^*^	12.8	(239)	^*^
Low job satisfaction	10.1	(6676)	12.4	(702)	^*^	10.4	(165)		12.5	(272)	^*^	13.7	(256)	^*^
Difficulty making ends meet	12.7	(8325)	23.6	(1315)	^*^	18.8	(295)	^*^	20.8	(445)	^*^	30.8	(561)	^*^
Low cash margin	14.9	(9793)	35.1	(1953)	^*^	22.4	(352)	^*^	29.0	(620)	^*^	52.7	(961)	^*^
Being socially active, mean (sd)	4.0	(2.3)	2.8	(2.3)	^*^	3.0	(2.2)	^*^	3.1	(2.4)	^*^	2.2	(2.1)	^*^
Low social support	9.9	(6448)	18.5	(1025)	^*^	15.6	(245)	^*^	14.9	(316)	^*^	25.2	(455)	^*^
Low practical support	3.6	(2333)	12.2	(684)	^*^	8.8	(139)	^*^	9.7	(208)	^*^	18.1	(331)	^*^
Low trust in others	22.1	(14446)	40.0	(2212)	^*^	28.6	(450)	^*^	42.1	(892)	^*^	46.9	(852)	^*^
Experiences of discrimination	19.8	(12972)	21.7	(1212)	^*^	19.0	(299)		22.5	(480)	^*^	22.9	(421)	^*^

^*^denotes a *p*-value < 0.05 between native-born and foreign-born; and separately between native-born and (1) Nordic, (2) European, and (3) non-European. Pearson’s chi-square and T-test

^a^Leave of absence, parental leave, taking care of one’s household, studying, training, disability pension, sickness benefits and long term sick leave

^b^Disposable income is shown in thousand (tkr) Swedish kronor (SEK) within quintile (Q) 1–5

Three different aspects of labour market integration were assessed; current labour market position, psychosocial working conditions and economic disparities. *Labour market position* was measured with 12 different position choices, which were then categorised into three groups: (1) employed/self-employed (reference), (2) unemployed/labour market measure, and (3) outside the labour market (including leave of absence or parental leave, studying or training, taking care of one’s household, sickness benefits, disability pension, and long term sick leave (more than 3 months)). Early retirements and written answers of other employment positions were excluded. Economic disparities were measured with variables that assessed participants’ *financial strain*, i.e. problems managing ongoing expenses, such as food, rent and bills during the past 12 months (no, one time or several occasions, dichotomised into yes or no) and *low cash margin*, i.e. inability to obtain 15,000 SEK (approx. 1600 EUR) in 1 week for an unforeseen situation (yes or no). Participants’ *psychosocial working conditions* were measured by three variables. The first two asked if the participants felt support and assistance from their *colleagues* and *closest manager* (4-point scale, dichotomised into ‘yes, mostly/to some extent’ and ‘no/do not know/do not have a manager’). The third variable asked whether the participants were *satisfied with their working duties* (5-point scale, dichotomised into ‘very satisfied /fairly satisfied’ and ‘neither satisfied nor unsatisfied/fairly unsatisfied/very unsatisfied’).

Social connections and networks were measured with items that assessed level of social activity, social- and practical support, and general trust in others. *Social activity* during the last 12 months was measured by assessing participation in 15 different activities, in areas such as courses and study circles, culture and sports events, political engagements, gatherings with family and friends and social networks online. The items (each with the response option yes or no) were added into a continuous index ranging from low to high social activity (0–15). *Social support* and *practical support* asked if participants had someone to share their inner feelings with (yes or no), and if they could receive practical help when needed (5-point scale, dichotomised into ‘yes/always or often’ and ‘no/never or mostly’). *Trust in others* asked if participants generally trusted other people (yes or no).

Discrimination experiences were assessed with an item that inquired if the participants had been treated unfairly or violated in the last 3 months (on a 3-point scale, dichotomised into yes, often/seldom or no). Participants were also asked if they could determine the reason for discrimination (such as ethnicity, religion, skin colour and appearance) but due to lack of impact, we only included general experiences of discrimination in the final models.

Six different items measured sociodemographic factors, based on register data. Participants’ *age* and *gender* measured as age during year of data collection (18 to 65 years old), and ‘men’ or ‘women’. *Occupational class* was based on Statistics Sweden’s socioeconomic classification [45] and coded into ‘blue-collar workers’ (unskilled manual workers and skilled manual workers) and ‘white-collar workers’ (assistant non-manual, intermediate non-manual, self-employed and other). *Income* was assessed using individual disposable income and coded into five quintiles. *Education* was coded as ‘compulsory education’, ‘secondary education’ and ‘post-secondary education’. *Civil status* was based on both register data and self-reported data of cohabiting living arrangements. The civil status variables were coded into ‘married/cohabiting’ and ‘unmarried/not cohabiting’ (including divorced and widow/widower).

### Analysis

Descriptive statistics were calculated with Pearson’s chi-square and t-tests to assess statistical differences between natives and migrants within the total sample, and between the migrant sub-samples by region of origin. The main analysis comprised a Blinder-Oaxaca decomposition for non-linear regression models [46, 47], with the aim to decompose the difference in mean psychological distress between natives and migrants across a set of explanatory factors. This is a data driven, explorative technique that enables us to assess multiple factors inherent in the complex phenomenon of social integration that can subsequently influence mental health. The method’s ability to quantify both the absolute and relative explained parts of inequalities has resulted in its increasing utilisation within fields such as gender-based income inequalities and socioeconomic health inequalities [47–50].

The analysis was conducted in two steps across four different paired groups; 1) between the native-born and all migrants in the total sample, and then between the native-born and migrants from 2) Nordic countries, 3) European countries and 4) non-European countries (using natives as reference category in each analysis). In the first step, the mean difference in psychological distress between each pair was calculated, while also taking into account group differences in the explanatory variables. In the second step, we estimated the magnitude of the health gap that could be explained by the observed group difference, and a detailed decomposition was applied to display each explanatory variable’s relative contribution to the health gap [51]. Positive values are interpreted as the average health improvement that could be expected in migrants if they had the same predicted values for the explanatory variables as the native-born. Negative values are instead interpreted as the potential increase in the mental health inequality between the paired groups. The latter is typically found when a factor that promotes health is over-represented in the advantaged group (e.g., a higher proportion of employment in the native-born) and is also related to factors which can negatively influence health among the disadvantaged group (e.g., a higher prevalence of educational mismatch or poor psychosocial working conditions in migrant groups) [52].

Estimations were reported as log odds and as the share of the relative contribution (presented as a percentage of the total explained contribution) of each explanatory variable to the mental health inequalities. The analyses were conducted in Stata 14, with the Oaxaca command (and the options of *logit* for non-linear decompositions, *pooled* model for group coefficients and *vcr* with 50 repetitions to estimate 95% Confidence Intervals). Survey year was also included in all analyses to adjust for potential calendar effects.

## Results

Descriptive statistics are presented in Table 1, showing that native-born persons on average had higher rates of employment and better psychosocial working conditions, fewer social and economic disadvantages and fewer experiences of discrimination than migrants. Similar social and economic patterns were found when dividing the foreign-born into Nordic, European and non-European groups, with Nordic migrants having the best outcomes among the foreign-born, followed by European and non-European migrants.

Psychological distress was also found to be more prevalent among migrants compared to natives (see Table 2). As a whole, the mean psychological distress score was 0.07 higher among migrants compared to natives (p < 0.05). When decomposing the mental health inequality, the most important explanatory social integration factor was social activity (0.014, p < 0.05, corresponding to 20% of the health gap). This finding can be interpreted as the expected mental health improvement among migrants if they had similar opportunities to engage in social activities as natives, which would decrease the mental health gap by 20%. Other important contributions were low trust in others (17%), financial strain (16%), low degree of social support (16%) and being outside the labour market (15%) or unemployed (10%). Having a low cash margin, low job satisfaction, lack of practical support and experiencing discrimination made smaller contributions (7–8%). Psychosocial working conditions and sociodemographic factors made low or non-significant contributions to the difference in psychological distress between natives and migrants. The findings further indicated that the total contribution of the social integration explanatory factors summed up to more than 100% (data not shown in table), suggesting that the mental health gap found was mainly due to within-group differences, thus highlighting the important influence of within-group variance.

**Table 2:** Non-linear Oaxaca decomposition of mental health (GHQ) gap between native-born and foreign-born in Sweden

GHQ foreign-born, mean (=n)	.38	(4496)
GHQ native-born, mean (=n)	.31	(60681)
Difference in GHQ	.07^*^	
Explanatory variables	Contribution to explained health gap (%)	
*Labour market position* ^a^		
Unemployed	.007^*^	10%
Outside the labour market	.011^*^	15%
*Economic hardship*		
Difficulty making ends meet	.012^*^	16%
Low cash margin	.006^*^	8%
*Psychosocial working conditions*		
Lack of support from co-workers	−.000	0%
Lack of support from manager	.000^*^	1%
Low job satisfaction	.005^*^	7%
*Social relations*		
Low social activity	.014^*^	20%
Low social support	.012^*^	16%
Low practical support	.005^*^	7%
Low trust in others	.012^*^	17%
Experiences of discrimination	.006^*^	8%
*Sociodemographic characteristics* ^a^		
Women	−.000	0%
Married/cohabiting	.001^*^	1%
Age	.000	0%
Blue-collar occupational class	−.001^*^	−1%
Income	.000	0%
Education		
Secondary education	−.001	−1%
Post-secondary education	.001^*^	2%

The results are presented as log odds (%). The relative contribution of each explanatory variables was calculated as the share (%) of the total difference in psychological distress

^*^denotes *p* < 0.05

^a^employed, men, not married or cohabiting, white-collar occupational class, compulsory education as reference categories

When dividing migrants by Nordic, European and non-European origin, a slightly different pattern was observed (see Table 3). Persons of Nordic origin reported on average a 0.05 higher psychological distress score compared to native-born persons (*p* < 0.05), whereas European and non-European populations reported psychological distress scores that were 0.08 higher than the native-born (p < 0.05). In analyses comparing natives and the Nordic population, lack of social activity (28%), social support (19%), being outside the labour market (18%) and financial strain (16%) were the largest explanatory factors that contributed to the mental health gap. Given equitable social integration in terms of social support, social engagements and paid employment, the mental health gap between natives and those of Nordic origin would be expected to decrease by around 80%. Other important factors were low trust in others, low practical support, age, sex, low cash margin and unemployment (4–11%).

**Table 3:** Decomposing mental health gap between native-born and Nordic-, European- and non-European persons in Sweden

	Nordic		European		non-European	
GHQ foreign-born, mean (=n)	.35	(1321)	.39	(1763)	.39	(1388)
GHQ native-born, mean (=n)	.31	(60681)	.31	(60681)	.31	(60681)
Difference in GHQ	.05^*^		.08^*^		.08^*^	
Explanatory variables	Contribution to explained health gap (%)		Contribution to explained health gap (%)		Contribution to explained health gap (%)	
*Labour market position* (Employed as reference)						
Unemployed	.002^*^	4%	.005^*^	6%	.014^*^	17%
Outside the labour market	.008^*^	18%	.007^*^	8%	.016^*^	19%
*Economic hardship*						
Difficulty making ends meet	.007^*^	16%	.009^*^	11%	.018^*^	21%
Low cash margin	.003^*^	6%	.004^*^	5%	.010^*^	12%
*Psychosocial working conditions*						
Lack of support from co-workers	.000	0%	.000	0%	−.000	0%
Lack of support from manager	−.001	−1%	.000	0%	.001^*^	1%
Low job satisfaction	.001	3%	.006^*^	7%	.006^*^	7%
*Social relations*						
Low social activity	.013^*^	28%	.011^*^	13%	.019^*^	22%
Low social support	.009^*^	19%	.008^*^	9%	.019^*^	22%
Low practical support	.003^*^	7%	.005^*^	5%	.009^*^	10%
Low trust in others	.005^*^	11%	.015^*^	17%	.016^*^	19%
Experiences of discrimination	−.000	−1%	.007^*^	9%	.008^*^	10%
*Sociodemographic characteristics*						
Sex (men as reference)	.002^*^	4%	−.001	−1%	−.001	−2%
Married/cohabiting	.000	0%	.001^*^	1%	.001^*^	1%
Age	.005^*^	10%	−.001^*^	−1%	−.003^*^	−4%
Occupational class	−.001	−1%	−.001	−1%	−.002^*^	−2%
Income	.000	0%	.000	0%	.000	0%
Compulsory education						
Secondary education	−.000	0%	−.000	0%	−.001	−1%
Post-secondary education	−.002^*^	−4%	.003^*^	4%	.001^*^	2%

Non-linear Oaxaca decomposition presented as log odds (%)). The relative contribution of each explanatory variable was calculated as the share (%) of the total difference in psychological distress

^*^denotes *p* < 0.05

The mental health gap between the native-born and European-born populations was largely explained by low trust in others (17%), low social activity (13%), difficulty making ends meet (11%), lack of social support (9%) and experiencing discrimination (9%). Factors that made significant but smaller contributions were being outside the labour market or unemployed, low job satisfaction, lack of practical support and having a post-secondary education (4–8%). Analyses comparing the native-born and non-European populations indicated that low participation in social activities (22%), low social support (22%), economic hardship (21 and 12%), low trust in others (19%), unemployment (19%) and being outside the labour market (17%) all made significant contributions to the mental health gap between these groups. Discrimination and lack of practical support each explained 10% of the health gap. The findings further indicated that being older and having a white-collar occupational class increased the health gap between groups; these factors were more prevalent among the native-born and were also related to poor mental health among the non-European population.

## Discussion

In the present study, we assessed for mental health inequalities between natives and different groups of migrants in Sweden, and examined the extent to which different social integration factors contributed to these inequalities. The findings showed that psychological distress was significantly more common among migrants from Nordic-, European- and non-European countries than among natives. Our findings further indicated that the predominant social integration indicators explaining the mental health inequalities were financial strain, lack of social activity and low social support. In stratified analyses, being unemployed and outside the labour market were also identified as important factors explaining the health gap between natives and the Nordic and non-European populations, while low trust in others and discrimination contributed to this gap in the European and non-European populations.

Prior research has demonstrated associations between poor mental health and different aspects of social integration [10, 11, 24, 32, 53], although only a paucity have used a multidimensional approach such as ours [32], which allows for a more holistic assessment of the relationship between social integration and mental health. Compared to the evidence on labour market integration [24, 53], previous public health research has paid less attention to social connections and networks, as well as the complex interplay between economic and social resources in social integration. Research on social relationships and health has shown that having access to social networks and social support decreases stress and buffers against mental ill health [8]. This may be of particular relevance for migrants, who typically have fewer friends and family in the host country and therefore also often have less access to social arenas than natives [10, 30]. Findings from this study emphasise that even when taking labour market factors into account, social connections and networks remained an important determinant of mental health inequality. In fact, lack of social support and engagement in social activities explained about one third of the mental health inequality between natives and migrants, and approximately 40% of the health gap between natives and Nordic and non-European migrants, respectively. Lack of practical and social support among migrants may entail a lack of social ties in everyday life, leading to feelings of social instability, social isolation or lack of belonging in the new society, which can negatively influence ability to adapt and cope with a new social environment and mental health [3, 31]. Conversely, participation in social and sports activities could buffer against mental ill health by increasing trust in other people. This in turn may facilitate cultural adaptation and coping abilities to handle post-migration stressors, such as discrimination, thereby also serving as a protective factor against poor mental health [11, 32]. This study therefore stresses the importance of social connections as a key component in migrants’ social integration and a determinant of migrant mental health. In addition, given the lack of studies that have simultaneously assessed labour market and social connectivity aspects of integration, the persistence of lack of social relations as a key contributor to mental health inequalities between natives and migrants might also suggest that lack of social network is an overlooked component of unemployment that may be particularly important for mental health; however, further studies are needed to assess this possibility.

Our findings are in line with previous research suggesting that financial strain is a key risk factor for poor mental health among migrants [24, 53]. This relationship persisted even after accounting for unemployment, being outside the labour market, psychosocial working conditions and sociodemographic factors. Financial hardships may have a stronger influence on mental health among persons of foreign-origin than among natives for several reasons. First, migrants may experience such hardships to a greater degree than natives due to delayed post-migration labour market entry and lower average earned wages than natives [54]. Migrants also tend to face greater insecurity in the labour market, which may result in periods of unemployment and resulting financial strain. In fact, prior studies have suggested that it can take up to 10 years before migrants in Sweden experience living conditions equal to those of the native-born [55]. Migrants may also lack the professional social networks available to natives that could assist with social mobility. In addition, many migrants send remittances to family members or friends in the country of origin [56] which entails less disposable income available in the country of residence. As such, migrants may continue to experience financial difficulties for extended periods post-migration. Persistent financial hardships may be one important contributing factor to the growing income inequality in Sweden [57] as well as the creation of marginalized groups characterized by economic hardship and ill health.

Although experiences of discrimination have previously been associated with poorer self-rated health and mental ill health, as well as poorer social integration [2, 36, 37], in the current study discrimination had a lower impact on the mental health inequality between migrants and natives than economic or other social aspects of integration. However, experiences of discrimination are likely to interact with other experiences of social and material adversity, such as social isolation and financial strain [58, 59], which similarly influence social integration and mental ill health. Further research is therefore needed to explore the combined influence of these factors on mental health.

### Limitations

This study contributes novel insights describing the extent to which different aspects of social integration can explain mental health inequalities between natives and different migrant groups in Sweden. Preforming such analysis requires rich and comprehensive data, such as the HET-surveys, which contain reliable self-reported and register based data compiled by Statistics Sweden [41]. However, one limitation of our research is the cross-sectional study design, which prevents us from making any causal inferences or controlling for previous health status. Another potential issue with self-reported data is the generally low response-rate in surveys and potential response-bias. That is, people with relatively poor health and lower levels of social integration may be more likely to decline participation in research studies, suggesting that the mental health disparities found in this study may be underestimated. However, the data collection and sensitivity analysis conducted by Statistics Sweden showed no systematic patterns in participant dropout rates, supporting the reliability of the findings.

The register-based measure of country of origin used in this study has the strength of having relatively high reliability in comparison with self-reported reason for migration, which has a greater potential for misclassification bias [60, 61]. However, we were unable to control for other factors that may influence social integration, such as language skills, neighbourhood segregation, as well as the nativity of the parents or grandparents of the native-born, which could bias our findings. The inability to account for the migration background of the native-born could entail that the mental health gap between the native- and foreign-born is even larger than estimated in the current [62]. Consequently, due to the complexity of capturing the multi-dimensional phenomenon of social integration, our findings should be interpreted with caution.

A limitation related to the non-linear Oaxaca–Blinder decomposition used in this study is the sensitivity to the reference group and the imputation order of explanatory variables [49], which can potentially influence the results. However, the selection of the foreign-born as the more socially and materially disadvantaged group has empirical support [10, 23, 30]. Robustness of the results was also tested using random imputation of variables into the models.

## Conclusion and policy implications

This study quantified the extent to which different aspects of social integration may explain mental health inequalities between natives and migrants in Sweden. Multiple aspects of social integration appeared to play a central role in affecting mental health among migrants in Sweden, with financial strain, social relations, and civic engagement standing out as the most important aspects of social integration. Among Nordic and non-European migrants, lack of labour market participation was identified as a particular barrier for social integration and mental health, while lack of trust and discrimination had an adverse effect on European and non-European migrants’ health and social integration. Future research and public health works should give greater consideration to migrants’ social networks and social support, which this study suggests may be just as important as labour market integration in influencing migrants’ mental health. Despite Sweden’s historically generous migration and integration polices, native-migrant inequalities in mental health were observed in the current study, particularly among migrants from non-European countries. However, the data for this study was conducted before the 2016 implementation of increasingly restrictive entry, right to residence, and integration policies in Sweden, which were a response to the large number of refugees and asylum seekers that arrived in the country in 2015. This development, in conjunction with an increasingly flexible and polarized labour market, suggests that newly arrived migrants may face increasing resettlement and integration difficulties, potentially leading to more severe mental health inequities between the native- and foreign-born.

## Abbreviations

GHQ: General Health Questionnaire
HET: Health on Equal Terms
